# Post-conditioning sleep deprivation facilitates delay and trace fear memory extinction

**DOI:** 10.1186/s13041-024-01163-w

**Published:** 2024-11-29

**Authors:** Daisuke Miyamoto, Mahmoud Abdelmouti Mahmoud

**Affiliations:** 1 Laboratory for Sleeping-Brain Dynamics, Research Center for Idling Brain Science, University of Toyama, 2630 Sugitani, Toyama, 930-0194 Japan; 2 Graduate School of Medicine and Pharmaceutical Sciences, University of Toyama, 2630 Sugitani, Toyama, 930-0194 Japan; 3 Department of Biochemistry, Faculty of Pharmacy, Cairo University, Cairo, 11562 Egypt

**Keywords:** Sleep deprivation, Learning & memory, Sex difference, Delay and trace auditory fear conditioning, Fear extinction and spontaneous recovery

## Abstract

Trace and delay auditory fear conditioning involve different memory association strategies based on working memory involvement; however, their differences in long-term processing through sleep and extinction training remain unclear. While females often exhibit more persistent fear, complicating psychiatric treatment, most studies have primarily focused on how sleep affects initial recall in male mice. We investigated the three-way interaction between tests (trace vs. delay), sleep states, and sex during initial recall, extinction, and post-extinction remote recall. A six-hour post-conditioning sleep deprivation (SD) did not affect freezing behavior during the following day’s extinction training of delay fear memory. However, during post-extinction remote recall of delay fear memory, SD prevented spontaneous recovery in males and reduced persistent freezing in females. In contrast, SD rapidly facilitated extinction of trace fear memory. In summary, SD enhances extinction both in the short-term and long-term, depending on the conditioning protocol. These findings highlight the importance of long-term assessments to explore interactions among emotional memory, sleep, and sex differences, with implications for individualized mechanisms underlying post-traumatic stress disorder (PTSD) and its treatments.

## Introduction

Newly acquired memories are consolidated during subsequent sleep for long-term storage [1, 2]. The role of sleep in memory consolidation has been explored through various behavioral tests in rodents. Studies show that sleep deprivation (SD) impairs memory consolidation in hippocampus-dependent tests, such as contextual fear conditioning and object-place recognition, but not in tests less reliant on the hippocampus, such as auditory delay fear conditioning and the novel object recognition test [3–5]. Recent studies, however, show that while newly formed memories in novel object recognition do not depend on hippocampal activity during sleep, remote memories do [6, 7]. Additionally, memory consolidation in auditory delay fear conditioning is influenced by the inactivation of prefrontal interneurons [8] but not by hippocampal theta waves during rapid-eye movement (REM) sleep [9]. These findings underscore the importance of considering multiple brain regions and time points to understand the relationships between sleep and memory.

Fear conditioning and extinction training are well-established rodent models for studying post-traumatic stress disorder (PTSD) and exposure therapy. Extinction training decreases learned fear through repeated, nonreinforced presentations of the conditioned stimulus (CS) without the aversive unconditioned stimulus (US). This process does not completely erase the original CS–US association but creates a safety memory that competes with the fear memory [10–12]. Persistent fear memories can overshadow safety memories, leading to spontaneous recovery of fear responses over time and complicating treatment efforts [13, 14]. PTSD prevalence is twice as high in females as in males [15], and numerous studies have demonstrated slower extinction in females, both in humans and animals [16–20]. Therefore, we aimed to reveal the long-term dynamics of fear memory and extinction in both sexes to enhance our understanding and treatment of psychiatric disorders.

Among behavioral tests in rodents, auditory cued fear conditioning is particularly advantageous for studying associative memory due to its precise temporal control between CS and US. The direct coupling of the CS and US in delay fear conditioning produces stronger freezing behavior [21]. However, natural fearful situations can include an interval between cue stimuli and imminent danger (e.g., rustling leaves prior to a predator’s approach). Similarly, in the phenomenology of PTSD, intrusive memories include not only stimuli present during the traumatic event but also those encountered shortly beforehand [22]. Trace fear conditioning, which requires working memory to link CS and US through an interval, involves more engagement of the hippocampus and prefrontal cortex [23–26]. Although the delay and trace fear conditioning undergo different processes, their long-term processing through sleep and extinction training remains unclear. We hypothesized that trace fear memory is more susceptible to sleep, as global inter-regional synchronization serves for internal information integration [27]. Here, we assessed interactions among test (delay vs. trace), state (sleep vs. SD), and sex in freezing behavior during initial recall, extinction, and post-extinction remote recall.

## Materials and methods

### Animals

Male and female C57BL/6J mice, aged 7 weeks, were purchased from SLC (Shizuoka, Japan). The mice were between 2 and 3 months old at the start of behavioral tests. Prior to the behavioral tests, they were singly housed for at least one week and handled by a male experimenter for three consecutive days. The animals were kept on a 12-hour light-dark cycle with food and water provided ad libitum. Home cages (155 × 245 × 148 mm, lwh) were stored in four ventilated soundproof boxes (60 × 70 × 60 cm, lwh) with staggered light-on times (9:30, 10:00, 10:30, or 11:00) to allow four consecutive behavioral experiments with four animals at the same circadian timing. Each behavioral session ended at Zeitgeber time (ZT0) in order to initiate post-conditioning 6-hour SD at the onset of the inactive period. SD was performed by gently touching the mice with a cotton swab during immobile timings, as visually detected by male and female experimenters.

### Delay fear conditioning

A black polyvinyl chloride behavioral chamber (166 × 146 × 238 mm, lwh) was placed inside a soundproof box (60 × 70 × 60 cm, lwh). The behavioral chamber was wiped with 70% ethanol after each behavioral session for each mouse. The same chamber was used for both delay and trace fear conditioning. Day 0 (Habituation): After a 2-minute baseline period in the chamber (20 lux lighting with cone bedding), 4 CS- (2 kHz) and 4 CS+ (10 kHz) tone (30 s, 75 dB) were presented with a pseudo-random inter-trial interval (45 to 90 s) in an intermingled manner. Day 1 (Conditioning): Following a 2-minute baseline period with a metal grid floor, 5 CS- and 5 CS+ were applied with a pseudo-random inter-trial interval of 45 to 90 s in an intermingled manner. At the end of CS+, a foot shock (0.5 s, 0.4 A) was delivered by a shock generator (Ohara & Co. Ltd., Tokyo, Japan). Day 2 (Extinction #1): After a 3-minute baseline period in the Habituation chamber, 4 CS- and then 12 CS+ were applied with a pseudo-random inter-trial interval of 45 to 90 s. Day 17 (Remote recall): After a 2-minute baseline period in the Habituation chamber, 4 CS- and the subsequent 4 CS+ were applied with a pseudo-random inter-trial interval of 45 to 90 s. Day 18 (Extinction #2): The same configuration as on Day 2.

### Trace fear conditioning

Day 0 (Habituation): Following a 2-minute baseline, 4 CS+ tone (10 kHz, 10 s, 75 dB) were presented with a 230-second inter-tone interval. Day 1 (Conditioning): After a 2-minute baseline, 5 CS+ followed by a 20-second trace period and a foot shock (0.5 s, 0.4 A) were presented with a 230-second inter-tone interval. Day 2 (Extinction #1): After a 3-minute baseline, 16 CS+ were presented with a pseudo-random inter-tone interval (65 to 110 s). Day 17 (Remote recall): The same configuration as on Day 0. Day 18 (Extinction #2): The same configuration as on Day 2.

### Data acquisition and analysis

The foot shock generator and tone function generator (OPR-SS2T, Ohara & Co. Ltd., Tokyo, Japan) were triggered by the OTPG-8 and the Fiberphotometry & Electrophysiology Console (Doric Lenses, Quebec, Canada). Animal motion was recorded via a webcam, and freezing scores were calculated using the Behavior Analysis module in Doric Neuroscience Studio. MATLAB was used for statistical analyses and freezing score conversion, with percentages calculated during the tone period for delay fear conditioning and both the cue and trace periods for trace fear conditioning.

A three-way analysis of variance (ANOVA) assessed freezing during the last three CS+ and three CS- trials on Day 1 for delay fear conditioning, the last three CS+ trials on Day 1 for trace fear conditioning, and the first four trials on Day 2 for both tests. The Day 1 trials were used for the covariate in analysis of covariance (ANCOVA). Additionally, a two-way ANOVA evaluated freezing during the last three CS+ trials on Day 1, the early and late six CS+ trials on Day 2 and Day 18, or all four trials on Day 17. Those trials from Day 2, Day 17, and Day 18 served as dependent variables in the ANCOVA.

## Results

### Auditory fear conditioning protocols and behavioral schedule

**Fig. 1:**
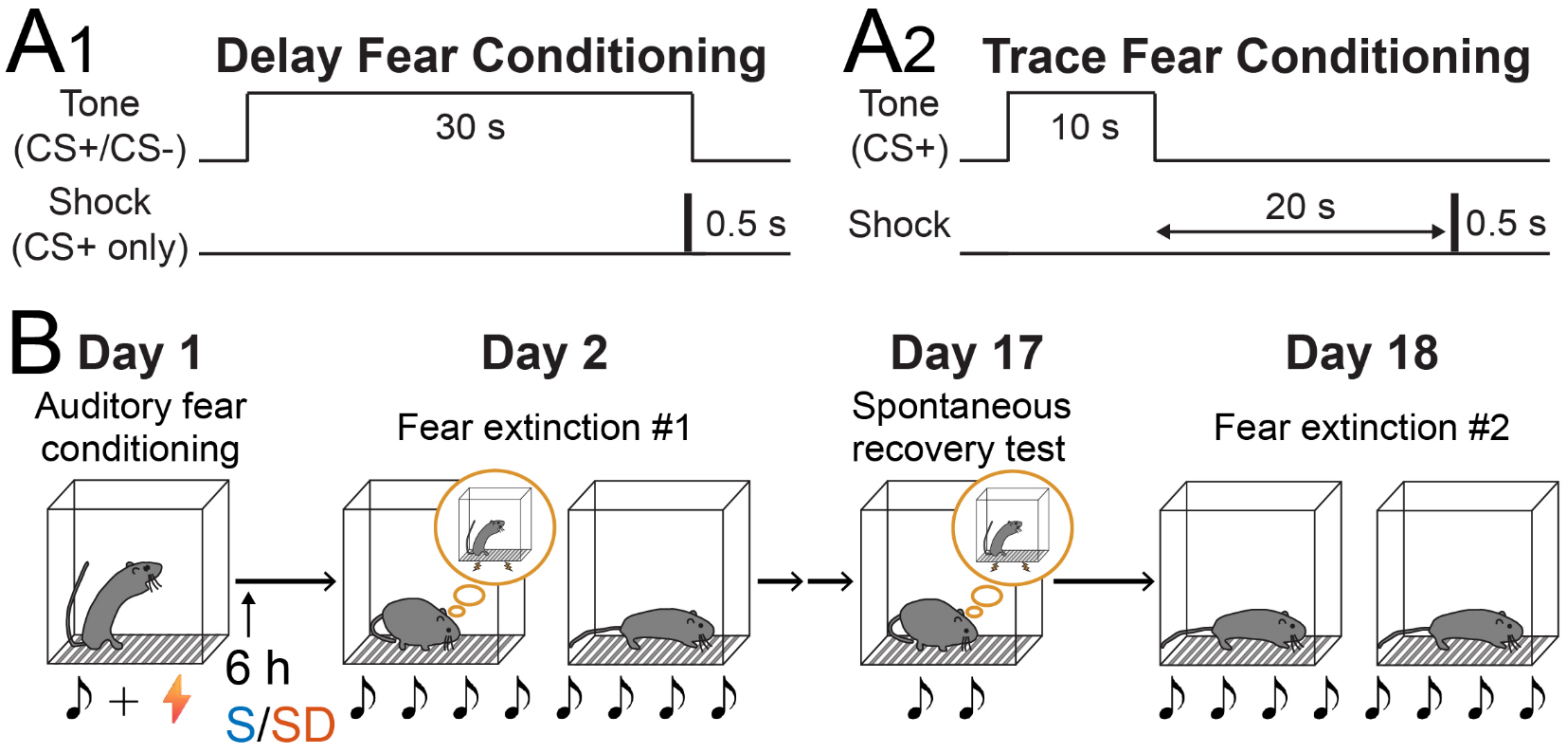
Protocols for delay and trace fear conditioning, followed by recall and extinction sessions. (**A**) In delay fear conditioning, an electrical foot shock was paired with the end of each CS+ (30 s, 10 kHz), while CS- (30 s, 2 kHz) was presented without a foot shock. In trace fear conditioning, the foot shock was delivered 20 s after the CS+ (30 s, 10 kHz). (**B**) Following auditory fear conditioning on Day 1, male and female mice were re-exposed to tone stimuli without the foot shock for Extinction #1 on Day 2, the Remote recall on Day 17, and Extinction #2 on Day 18. A subset of mice underwent 6-hour SD after auditory fear conditioning

Auditory stimuli were paired with electrical shocks in both delay and trace fear conditioning (Fig. 1A). In delay fear conditioning (Fig. 1A_1_), the paired conditioned stimulus (CS+) was followed by an electrical shock at its termination, while the unpaired conditioned stimulus (CS-) was not. In trace fear conditioning (Fig. 1A_2_), the CS+ was followed by a shock after a 20-second trace interval. Mice underwent either delay or trace fear conditioning on Day 1, followed by re-exposure to the auditory stimuli during Extinction #1 on Day 2, Remote recall on Day 17, and Extinction #2 on Day 18 (Fig. 1B). After conditioning, mice were either allowed to sleep (S) or subjected to 6-hour SD.

### Higher initial memory retrieval in delay fear conditioning compared to trace fear conditioning, and in females compared to males

**Fig. 2:**
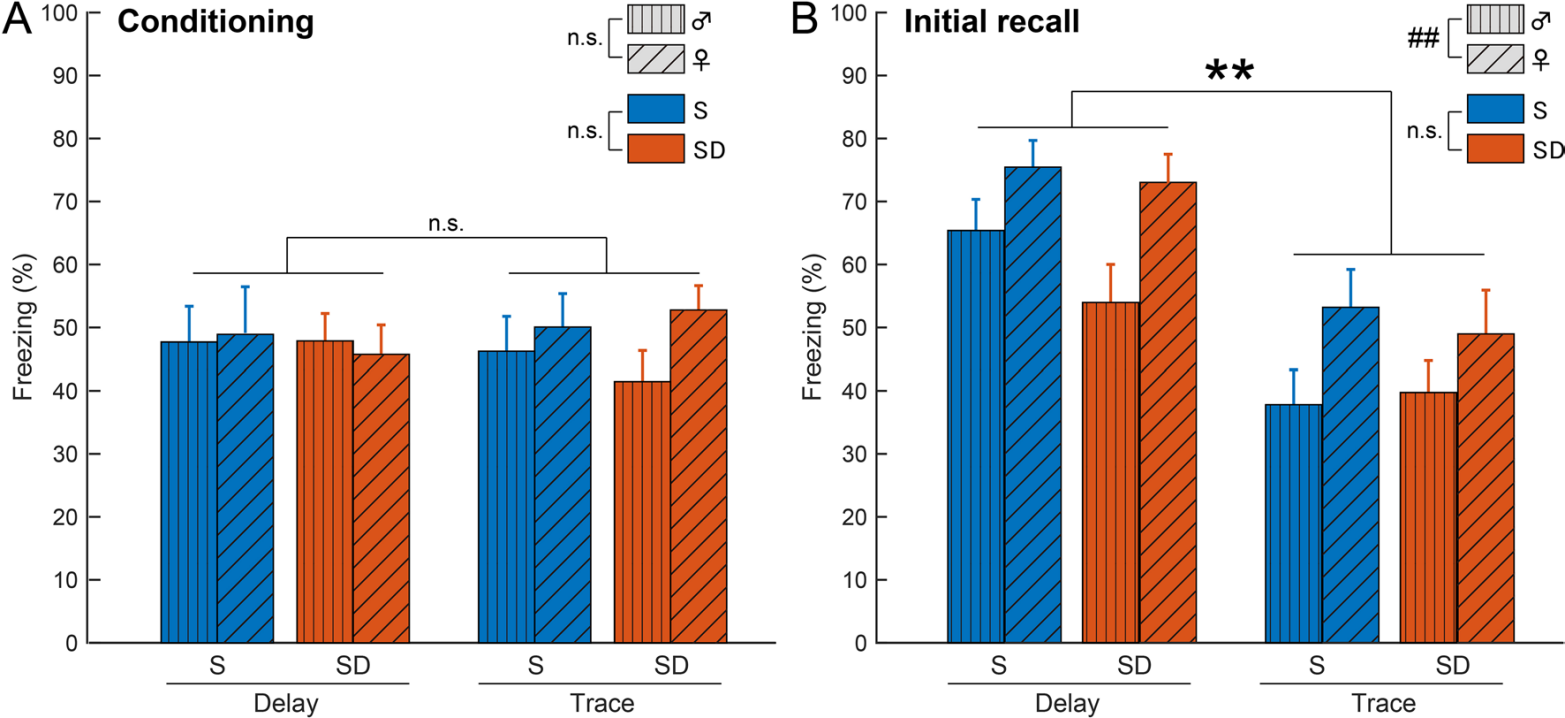
Higher freezing in initial recall in delay fear conditioning and in females. (**A**) During the last trials on Day 1, no significant differences were observed across test, sex, or state. (**B**) During the initial trials on Day 2, higher freezing levels were observed in the delay fear conditioning group and in females (**p < 0.01 for the main effect of the test factor, ##*p* < 0.01 for the main effect of the sex factor, three-way ANOVA, Table 1). Sample sizes for the delay fear conditioning group were *n* = 10 for male S, *n* = 10 for male SD, *n* = 9 for female S, and *n* = 10 for female SD mice. In the trace fear conditioning group, sample sizes were *n* = 10 for male S, *n* = 10 for male SD, *n* = 10 for female S, and *n* = 9 for female SD mice. Values represent the mean ± standard error of the mean (SEM)

We examined whether sex (male vs. female), test (delay vs. trace fear conditioning), or state (S vs. SD) influenced memory acquisition and initial recall. A three-way ANOVA (Table 1) on the last trials of Day 1 found no significant main effects for sex, test, or state, nor any interactions between these factors (Fig. 2A). However, during initial trials on Day 2, significant main effects were found for sex (*F* (1, 70) = 12.27, *p* = 0.0008) and test (*F* (1, 70) = 32.44, *p* < 0.0001), with no main effects of state or interactions (Fig. 2B). This indicates that females and mice in the delay conditioning exhibited higher freezing during initial fear memory recall, which was not affected by post-conditioning SD. We then examined the time course of freezing during recall and extinction sessions for each test and sex.

**Table 1:** Three-way ANOVA results for the last trials on day 1 and the initial trials on day 2

	Figure 2A: Conditioning	Figure 2B: Initial recall
Main Effect of Sex	*F* (1, 70) = 0.42, *p* = 0.5213	*F* (1, 70) = 12.27, *p* = 0.0008
Main Effect of State	*F* (1, 70) = 0.13, *p* = 0.717	*F* (1, 70) = 1.09, *p* = 0.3006
Main Effect of Test	*F* (1, 70) = 1.47, *p* = 0.2292	*F* (1, 70) = 32.44, *p* < 0.0001
Sex x State Interaction	*F* (1, 70) = 0.16, *p* = 0.6874	*F* (1, 70) = 0.03, *p* = 0.8552
Sex x Test Interaction	*F* (1, 70) = 1.96, *p* = 0.1662	*F* (1, 70) = 0.08, *p* = 0.7725
State x Test Interaction	*F* (1, 70) < 0.01, *p* = 0.9464	*F* (1, 70) = 0.60, *p* = 0.4422

The main effects of sex, state, and test, as well as interactions between them, are reported for the last trials on Day 1 (Fig. 2A) and the initial trials on Day 2 (Fig. 2B)

### Post-conditioning SD enhanced gradual fear extinction of delay fear memory

**Fig. 3:**
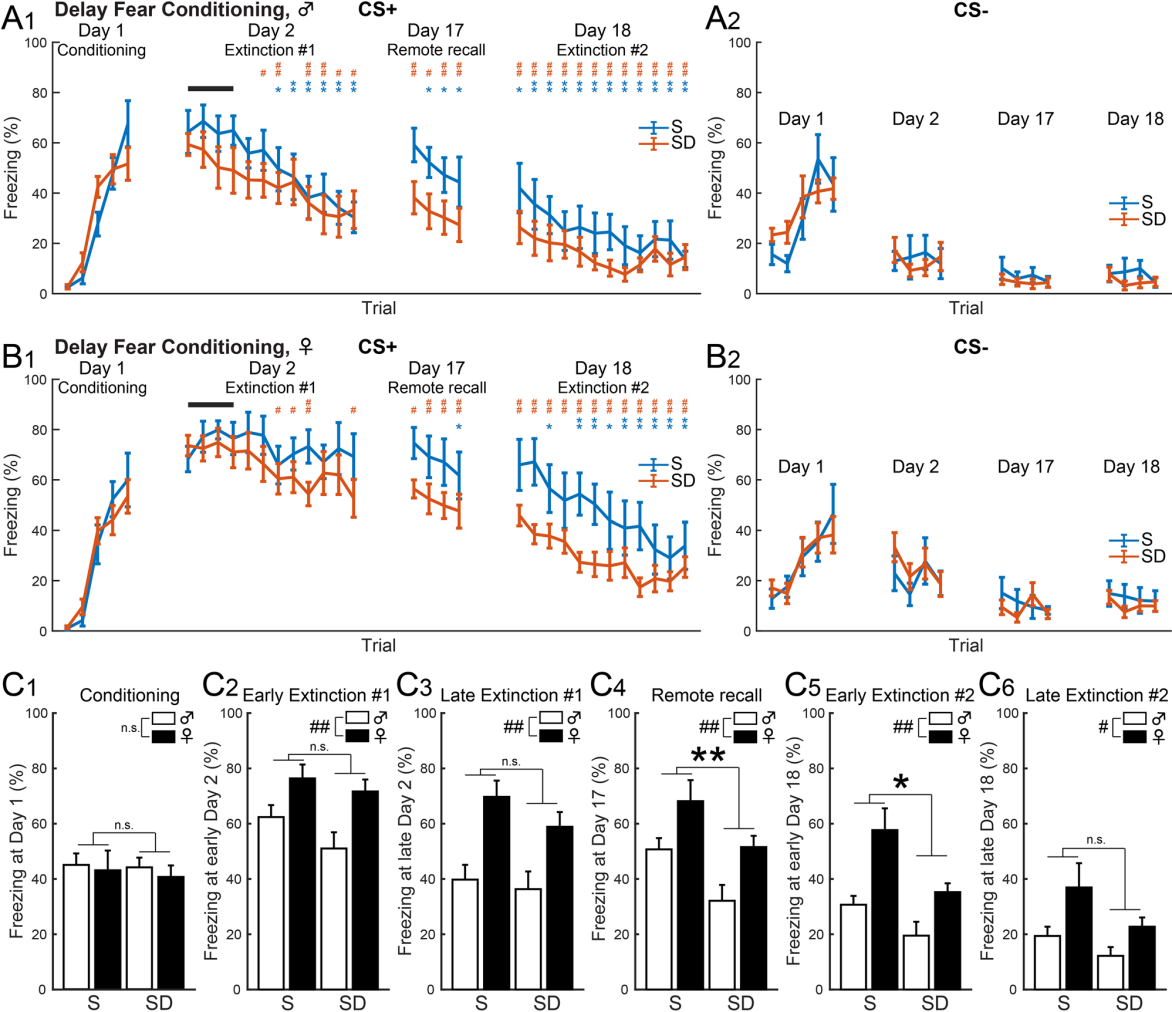
In delay fear conditioning, post-conditioning SD enhanced gradual fear extinction. (**A**_1_) Male S mice (*n* = 10) showed decreased freezing during CS+ from Day 2, followed by Remote recall on Day 17 and re-extinction on Day 18. Male SD mice (*n* = 10) decreased freezing during CS+ on Day 2 without spontaneous recovery on Day 17 (*p < 0.05 and **p < 0.01 for S mice, #*p* < 0.05 and ##*p* < 0.01 for SD mice, multiple paired *t*-tests vs. initial 4 trials on Day 2). (**A**_2_) CS- triggered low-level freezing in male S and SD mice. (**B**_1_) Female S mice (*n* = 9) demonstrated decreased freezing during CS+ from Day 17. Female SD mice (*n* = 10) decreased freezing during CS+ from late trials on Day 2 without spontaneous recovery on Day 17. (*p < 0.05 and **p < 0.01 for S mice, #*p* < 0.05 and ##*p* < 0.01 for SD mice, multiple paired *t*-tests versus initial 4 trials on Day 2). (**B**_2_) CS- triggered low-level freezing in female S and SD mice. (**C**_1_) Similar freezing during CS+ was observed across sexes and states on Day 1. (**C**_2 − 6_) Female mice showed higher freezing during CS+ across early (**C**_2_) and late (**C**_3_) Day 2, Day 17 (**C**_4_), and early (**C**_5_) and late (**C**_6_) Day 18. SD reduced freezing during CS+ on Day 17 (**C**_4_) and early Day 18 (**C**_5_). (*p < 0.05 and **p < 0.01 for the main effect of the state factor, #*p* < 0.05 and ##*p* < 0.01 for the main effect of the sex factor, two-way ANOVA, Table 2). Values represent the mean ± SEM

In males, both the S (*n* = 10) and SD (*n* = 10) groups exhibited increased freezing in response to both CS+ (Fig. 3A_1_) and CS- (Fig. 3A_2_) during delay fear conditioning on Day 1. During Extinction #1 on Day 2, both groups showed a significant decrease in freezing; however, only the S group restored high freezing levels during the Remote recall on Day 17, comparable to their initial recall on Day 2 (Fig. 3A_1_). Both male S and SD groups further reduced freezing to CS+ during Extinction #2 on Day 18 (Fig. 3A_1_). Additionally, low-level freezing was observed upon re-exposure to CS- on Day 2, 17, and 18 (Fig. 3A_2_), suggesting that SD did not affect motor functions or general fear or anxiety in male mice.

In females, both the S (*n* = 9) and SD (*n* = 10) groups showed increased freezing to CS+ (Fig. 3B_1_) and CS- (Fig. 3B_2_) during delay fear conditioning on Day 1. During Extinction #1 on Day 2, neither group showed significant decreases in freezing, with only mild reductions noted in the SD group (Fig. 3B_1_). In contrast, the SD group, but not the S group, exhibited significant reductions in freezing during post-extinction remote recall on Day 17 and in the early trials of Day 18. Unlike male S group (Fig. 3A_1_), the female S and SD groups did not show increased freezing from Day 2 to 17, potentially due to less effective extinction training with persistently high freezing on Day 2 (Fig. 3B_1_). As in males, low freezing responses to CS- were observed on Day 2, 17, and 18 in both female S and SD groups (Fig. 3B_2_).

A two-way ANOVA (Table 2) showed significant main effects of sex across early (Fig. 3C_2_, *F* (1, 35) = 11.61, *p* = 0.0017) and late (Fig. 3C_3_, *F* (1, 35) = 18.84, *p* = 0.0001) trials on Day 2, Day 17 (Fig. 3C_4_, *F* (1, 35) = 9.37, *p* = 0.0042), early (Fig. 3C_5_, *F* (1, 35) = 11.93, *p* = 0.0015) and late (Fig. 3C_6_, *F* (1, 35) = 6.36, *p* = 0.0164) trials on Day 18. Significant main effects of state were observed on Day 17 (Fig. 3C_4_, *F* (1, 35) = 8.56, *p* = 0.006) and early Day 18 (Fig. 3C_5_, *F* (1, 35) = 7.4, *p* = 0.0101). These results indicate that while sex influenced freezing during each set of trials for delay fear memory recall, post-conditioning SD reduced freezing during post-extinction remote recall in both males and females.

**Table 2:** Two-way ANOVA results for delay and trace fear conditioning

Figure	Task	Trials	Main Effect of Sex	Main Effect of State	Sex x State Interaction
Figure 3C1	Delay	Conditioning	*F* (1, 35) = 0.25, *p* = 0.6207	*F* (1, 35) = 0.09, *p* = 0.7637	*F* (1, 35) = 0.02, *p* = 0.8896
Figure 3C2	Delay	Early Extinction #1	*F* (1, 35) = 11.61, *p* = 0.0017	*F* (1, 35) = 2.52, *p* = 0.1217	*F* (1, 35) = 0.43, *p* = 0.5179
Figure 3C3	Delay	Late Extinction #1	*F* (1, 35) = 18.84, *p* = 0.0001	*F* (1, 35) = 1.39, *p* = 0.2456	*F* (1, 35) = 0.37, *p* = 0.5444
Figure 3C4	Delay	Remote Recall	*F* (1, 35) = 9.37, *p* = 0.0042	*F* (1, 35) = 8.56, *p* = 0.006	*F* (1, 35) = 0.03, *p* = 0.8694
Figure 3C5	Delay	Early Extinction #2	*F* (1, 35) = 11.93, *p* = 0.0015	*F* (1, 35) = 7.4, *p* = 0.0101	*F* (1, 35) = 0.83, *p* = 0.3682
Figure 3C6	Delay	Late Extinction #2	*F* (1, 35) = 6.36, *p* = 0.0164	*F* (1, 35) = 3.7, *p* = 0.0627	*F* (1, 35) = 0.39, *p* = 0.5345
Figure 4B1	Trace	Conditioning	*F* (1, 35) = 2.29, *p* = 0.139	*F* (1, 35) = 0.04, *p* = 0.8381	*F* (1, 35) = 0.56, *p* = 0.4607
Figure 4B2	Trace	Early Extinction #1	*F* (1, 35) = 3.79, *p* = 0.0596	*F* (1, 35) = 0.21, *p* = 0.6514	*F* (1, 35) = 0.44, *p* = 0.5135
Figure 4B3	Trace	Late Extinction #1	*F* (1, 35) = 0.47, *p* = 0.4968	*F* (1, 35) = 4.86, *p* = 0.0341	*F* (1, 35) = 0.75, *p* = 0.3929
Figure 4B4	Trace	Remote Recall	*F* (1, 35) = 0.43, *p* = 0.5164	*F* (1, 35) = 3.58, *p* = 0.0668	*F* (1, 35) = 0.66, *p* = 0.4217
Figure 4B5	Trace	Early Extinction #2	*F* (1, 35) = 0.38, *p* = 0.5398	*F* (1, 35) = 0.55, *p* = 0.4613	*F* (1, 35) = 1.98, *p* = 0.1682
Figure 4B6	Trace	Late Extinction #2	*F* (1, 35) = 0.01, *p* = 0.9181	*F* (1, 35) = 0.00, *p* = 0.9634	*F* (1, 35) = 2.09, *p* = 0.157

**Fig. 4:**
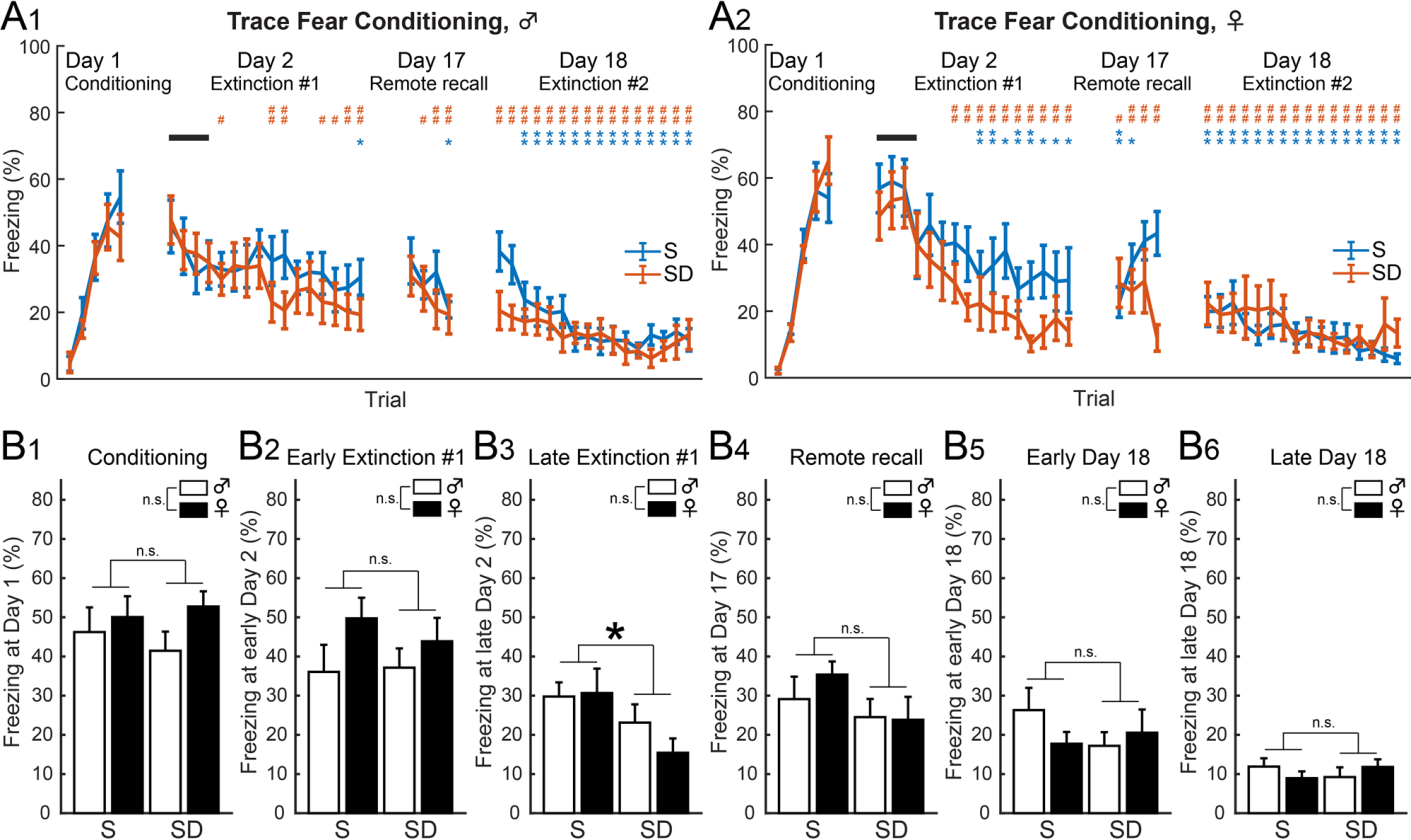
In trace fear conditioning, post-conditioning SD accelerated extinction. (A_1_) Male S mice (*n* = 10) showed mild decreases in freezing on Day 2 and strong decreases on Day 18. Male SD mice (*n* = 10) exhibited decreased freezing on Day 2 with further decreases on Day 18 (*p < 0.05 and **p < 0.01 for S mice, #*p* < 0.05 and ##*p* < 0.01 for SD mice, multiple paired *t*-tests vs. initial 4 trials on Day 2). (A_2_) Female S mice (*n* = 10) decreased freezing from Day 2 with further decreases on Day 18. Female SD mice (*n* = 9) showed strong decreases in freezing on Day 2 to levels comparable to Day 18. (*p < 0.05 and **p < 0.01 for S mice, #*p* < 0.05 and ##*p* < 0.01 for SD mice, multiple paired *t*-tests vs. initial 4 trials on Day 2). (**B**) SD decreased freezing on later Day 2 (B_3_) with no significant sex differences from Day 1 (B_1_), early (B_2_) and late (B_3_) Day 2, Day 17 (B_4_), to early (B_5_) and late (B_6_) Day18. (*p < 0.05 for state factor, two-way ANOVA, Table 2). Values represent the mean ± SEM

The main effects of sex, state, and interaction between them are reported for each set of trials (Conditioning, early and late Extinction #1, Remote recall, early and late Extinction #2) in delay (Fig. 3C) and trace fear conditioning (Fig. 4B)

### Post-conditioning SD accelerated trace fear extinction

Male S mice (*n* = 10) showed mild reductions in freezing on Day 2 and 17, with a more substantial decrease on Day 18 (Fig. 4A_1_). Male SD mice (*n* = 10), however, exhibited significant reductions on Day 2 and 17, with further reductions on Day 18. In females, while both S (*n* = 10) and SD (*n* = 9) mice began to decrease freezing on Day 2, with the SD group showing a more pronounced reduction (Fig. 4A_2_). A two-way ANOVA (Table 2) revealed significant main effects of state at late Day 2 (Fig. 4B_3_, *F* (1, 35) = 4.86, *p* = 0.0341), but no significant main effects of sex or interactions between state and sex. Thus, post-conditioning SD accelerated extinction of trace fear memory (Fig. 4B_3_) at an earlier stage than delay fear memory (Fig. 3C_4, 5_).

### Controlling for inter-individual variability: ANCOVA results

Freezing during fear conditioning and recall is influenced by variability arising from individual differences in responsiveness to neutral and aversive stimuli, associative learning ability, defense strategies such as freeze or flight, among other factors. In females, this variability can also be affected by the estrous cycle. To account for these variations, we performed an analysis of covariance (ANCOVA), using freezing on Day 1 as a covariate, to isolate the effects of SD on recall and extinction while controlling for differences in memory acquisition.

**Fig. 5:**
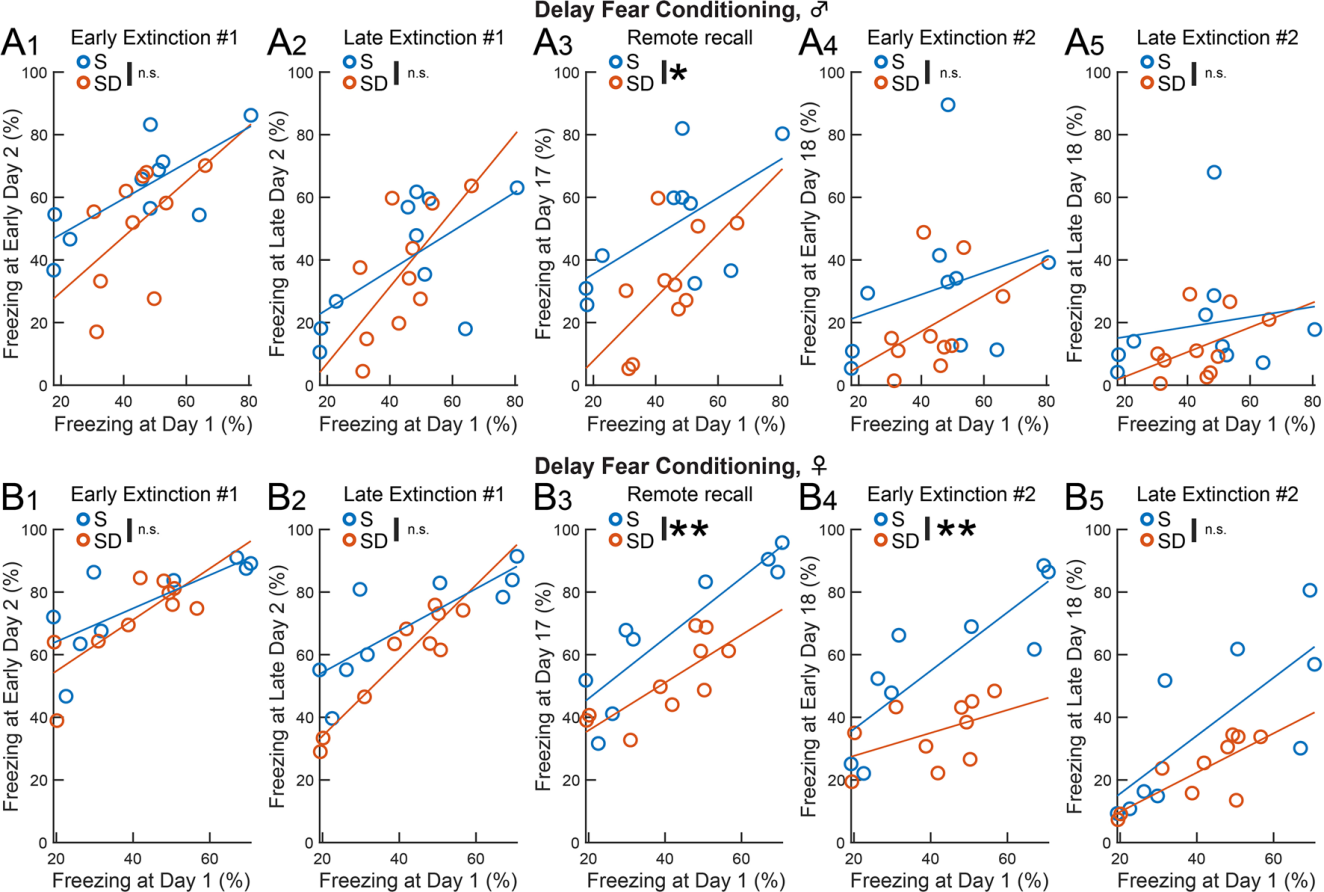
Post-conditioning SD gradually enhanced delay fear extinction, independent of pre-existing group differences. (**A**, **B**) Scatter plots and linear regression lines representing freezing behavior in male (**A**) and female (**B**) mice during each set of CS+ recall trials compared to conditioning on Day 1. Group differences between S and SD were tested while accounting for freezing on Day 1 as a covariate (ANCOVA, *p < 0.05, **p < 0.01, Table 3). Each set of re-exposure corresponds to early Day 2 (A_1_, B_1_), late Day 2 (A_2_, B_2_), Day 17 (A_3_, B_3_), early Day 18 (A_4_, B_4_), and late Day 18 (A_5_, B_5_). SD decreased freezing during Remote recall in male mice (A_3_) and during Remote recall (B_3_) and early Extinction #2 (B_4_) in female mice

**Fig. 6:**
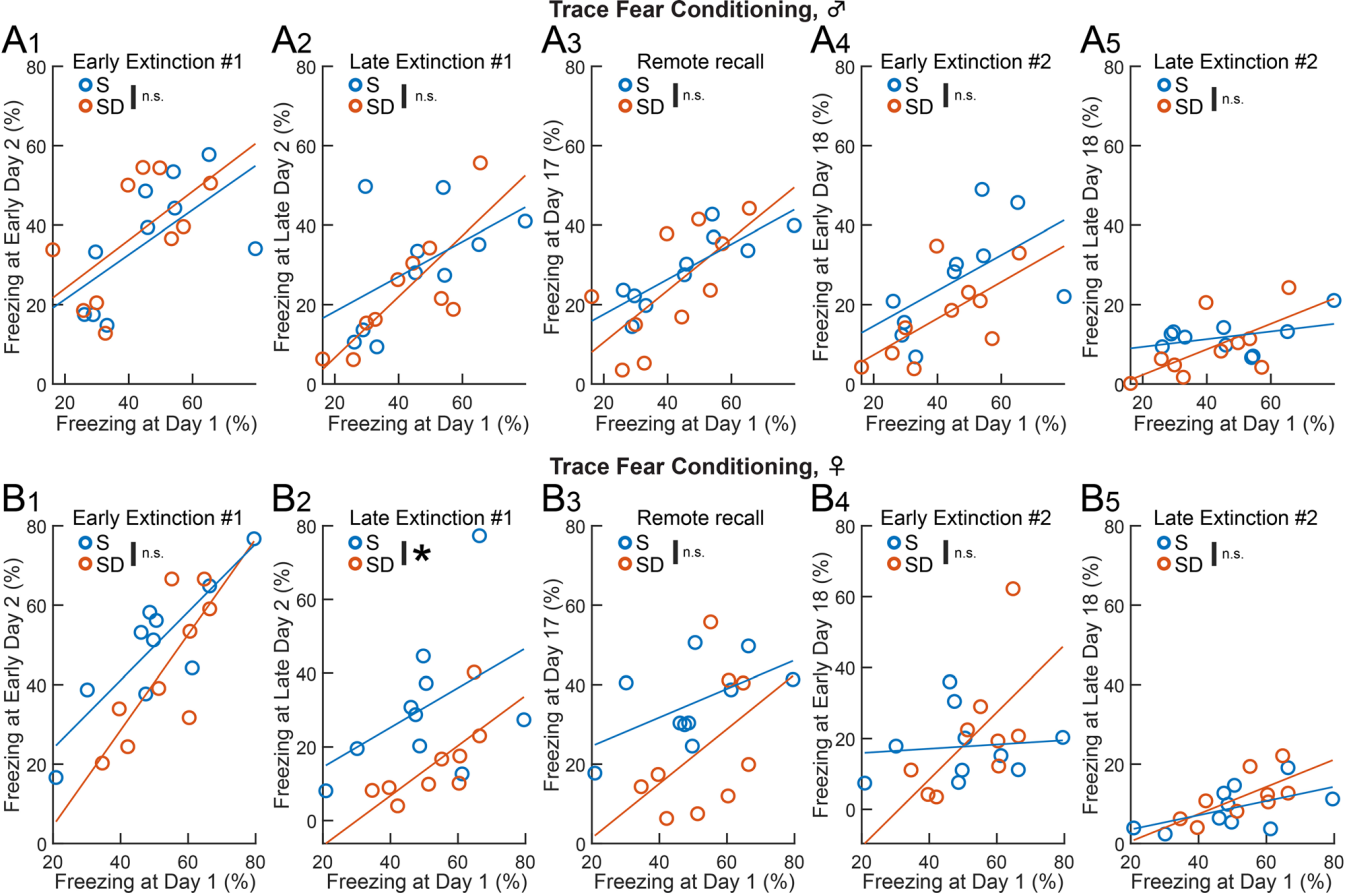
Post-conditioning SD accelerated trace fear extinction, independent of pre-existing group differences. (**A**, **B**) Scatter plots and linear regression lines representing freezing behavior in male (**A**) and female (**B**) mice during each set of CS+ recall trials compared to conditioning on Day 1. Group differences between S and SD were tested while accounting for freezing on Day 1 as a covariate (ANCOVA, *p < 0.05, Table 3). Each set of recall trials corresponds to early Day 2 (A_1_, B_1_), late Day 2 (A_2_, B_2_), Day 17 (A_3_, B_3_), early Day 18 (A_4_, B_4_), and late Day 18 (A_5_, B_5_). SD decreased freezing during late Extinction #21 in female mice (B_2_)

In delay fear conditioning, ANCOVA (Table 3) revealed that SD significantly reduced freezing on Day 17 in male mice (Fig. 5A_3_, *F* (1, 17) = 8.12, *p* = 0.022), and on Day 17 (Fig. 5B_3_, *F* (1, 16) = 31.54, *p* = 0.004) and early Day 18 (Fig. 5B_4_, *F* (1, 16) = 18.71, *p* = 0.002) in females. In trace fear conditioning, SD significantly decreased freezing in females on late Day 2 (Fig. 6B_2_, *F* (1, 16) = 5.70, *p* = 0.021). The positive slope of all linear regression lines (Figs. 5 and 6) indicates that inter-individual variability was maintained across sessions. Thus, the enhancement of extinction in both delay and trace fear memory by post-conditioning SD is not due to differences in individual variability during memory acquisition.

**Table 3:** ANCOVA results for freezing behavior in recall sessions

Figure	Task	Sex	Trials	F (df)	*p*-value
Figure 5A1	Delay	Male	Early Extinction #1	*F* (1, 17) = 6.76	0.102
Figure 5A2	Delay	Male	Late Extinction #1	*F* (1, 17) = 5.27	0.712
Figure 5A3	Delay	Male	Remote Recall	*F* (1, 17) = 8.12	0.022
Figure 5A4	Delay	Male	Early Extinction #2	*F* (1, 17) = 1.73	0.246
Figure 5A5	Delay	Male	Late Extinction #2	*F* (1, 17) = 1.08	0.308
Figure 5B1	Delay	Female	Early Extinction #1	*F* (1, 16) = 11.25	0.475
Figure 5B2	Delay	Female	Late Extinction #1	*F* (1, 16) = 22.86	0.061
Figure 5B3	Delay	Female	Remote Recall	*F* (1, 16) = 31.54	0.004
Figure 5B4	Delay	Female	Early Extinction #2	*F* (1, 16) = 18.71	0.002
Figure 5B5	Delay	Female	Late Extinction #2	*F* (1, 16) = 12.73	0.067
Figure 6A1	Trace	Male	Early Extinction #1	*F* (1, 17) = 5.34	0.506
Figure 6A2	Trace	Male	Late Extinction #1	*F* (1, 17) = 7.03	0.469
Figure 6A3	Trace	Male	Remote Recall	*F* (1, 17) = 9.77	0.611
Figure 6A4	Trace	Male	Early Extinction #2	*F* (1, 17) = 6.82	0.149
Figure 6A5	Trace	Male	Late Extinction #2	*F* (1, 17) = 3.64	0.493
Figure 6B1	Trace	Female	Early Extinction #1	*F* (1, 16) = 16.41	0.095
Figure 6B2	Trace	Female	Late Extinction #1	*F* (1, 16) = 5.70	0.021
Figure 6B3	Trace	Female	Remote Recall	*F* (1, 16) = 4.06	0.05
Figure 6B4	Trace	Female	Early Extinction #2	*F* (1, 16) = 1.12	0.762
Figure 6B5	Trace	Female	Late Extinction #2	*F* (1, 16) = 5.08	0.318

Statistics for group differences between S and SD in recall sessions (early and late Extinction #1, Remote recall, early and late Extinction #2) using freezing behavior in acquisition as a covariate in delay (Fig. 5A: male, Fig. 5B: female) and trace fear conditioning (Fig. 6A: male, 6B: female)

## Discussion

### Post-conditioning SD enhances early trace fear extinction and gradual delay fear extinction

Across both sexes and test protocols in auditory fear conditioning, we consistently found that post-conditioning SD preserved initial recall (Fig. 2B) but reduced freezing during later recall. This consistent reduction may be attributed to our focus on post-conditioning periods for fear memory consolidation, as post-extinction periods likely involve combined processes such as fear memory reconsolidation and extinction memory consolidation. SD effects emerged during trace fear extinction (Fig. 4B_3_) and after delay fear extinction (Fig. 3C_4_, 3C_5_), indicating that SD makes fear memory more susceptible to extinction in a test- and timing-dependent manner (Table 4). While the rapid facilitation of Extinction #1 by SD in trace fear conditioning is not attributed to impairment of reconsolidation, the reduction in freezing by SD after Extinction #1 in delay fear conditioning may reflect impairment of reconsolidation circuits, facilitation of extinction circuits, or both. From these findings and speculation, it seems that SD influences multi-regional processing necessary for recent trace and remote delay fear memory.

Different brain mechanisms underlie the distinctions between delay and trace fear memory in both conditioning and extinction. Trace fear memory requires working memory and awareness [28], necessitating greater involvement of the prefrontal cortex and hippocampus during conditioning [23]. During extinction, while the infralimbic cortex is activated in both tests, the amygdala is necessary for delay fear memory extinction, whereas the retrosplenial cortex is required for trace fear memory extinction [29]. Furthermore, emotional memory circuits, including the prefrontal cortex, hippocampus, thalamus, and amygdala, communicate inter-regionally while establishing fear and extinction memory engrams [30]. In remote memory, neural coding can drift within each brain region [31] and shift across brain regions through systems consolidation [32, 33]. Besides neural activity, the remote effects of SD in extinction recall may also be attributed to sleep-dependent long-term inter-regional synaptic plasticity and relevant molecules [34]. To address dynamic and heterogeneous neural populations and their interactions across long-time course, we have constructed fiber photometry systems targeting multiple brain regions with cell-type specificity and sleep-state resolution [35–37].

In addition to the possible qualitative differences in brain mechanisms, a quantitative difference also underlies the distinction between delay and trace fear memory. Although mice show higher freezing during initial recall of delay fear memory (Fig. 2B), the test-dependent SD effects are unlikely to be due to quantitative differences, as the freezing levels during initial recall are similar between males in delay fear memory and females in trace fear memory. Additionally, SD consistently decreased freezing during post-extinction remote recall in males and females, who exhibited different freezing levels across the sets of recall trials (Fig. 3C_2 − 6_). While we consistently used 5 CS+–US association trials across tests and sexes, adjusting this number (e.g. 4 trials for delay vs. 6 trials for trace) may help align freezing levels [38].

**Table 4:** Timeline summary of freezing decrease by SD in delay and trace fear conditioning

		Conditioning	Initial recall	Early Extinction #1	Late Extinction #1	Remote recall	Early Extinction #2	Late Extinction #2
Delay	Figures	2 A, 3C_1_	2B	3C_2_, 5A_1_ (♂), 5B_1_ (♀)	3C_3_, 5A_2_ (♂), 5B_2_ (♀)	3C_4_, 5A_3_ (♂), 5B_3_ (♀)	3C_5_, 5A_4_ (♂), 5B_4_ (♀)	3C_6_, 5A_5_ (♂), 5B_5_ (♀)
	SD effects	n.s.	n.s.	n.s.	n.s.	*p* < 0.01 for 3C_4_ (both sex) and 5B_3_ (♀), *p* < 0.05 for 5A_3_ (♂)	*p* < 0.01 for 5B_4_ (♀), *p* < 0.05 for 3C_5_ (both sex)	n.s.
Trace	Figures	2 A, 4B_1_	2B	4B_2_, 6A_1_ (♂), 6B_1_ (♀)	4B_3_, 6A_2_ (♂), 6B_2_ (♀)	4B_4_, 6A_3_ (♂), 6B_3_ (♀)	4B_5_, 6A_4_ (♂), 6B_4_ (♀)	4B_6_, 6A_5_ (♂), 6B_5_ (♀)
	SD effects	n.s.	n.s.	n.s.	*p* < 0.05 for 4B_3_ (both sex) and 6B_2_ (♀)	n.s.	n.s.	n.s.

Summary of figure panels and significant effects of SD on freezing responses across trial sets in delay and trace fear conditioning. SD reduced freezing responses during Remote recall and early Extinction #2 in delay fear conditioning, and during late Extinction #1 in trace fear conditioning

### Sex differences in recall and extinction

In delay fear extinction, male mice decreased freezing during Extinction #1 and recovered freezing in Remote recall (Fig. 3A_1_), while females showed persistently high levels of freezing (Fig. 3B_1_). These sex differences align with previous reports and have been linked to neural activity in various brain regions, including the prefrontal cortex, insular cortex, hippocampus, amygdala, and hypothalamus, all of which have receptors for sex hormones [39]. Among sex hormones, estradiol administration facilitates extinction in ovariectomized female rats [40]. Also, female rats in the proestrus phase during extinction (Day 2) have been reported to exhibit lower freezing across conditioning (Day 1), extinction (Day 2), and test (Day 3) compared to those in the metestrus phase [21]. Many studies have used delay fear conditioning for testing sex differences or estrous cycle dependency, some studies showed higher freezing in females in trace fear memory [41], which is consistent to our study (Fig. 2B), and acquisition of trace fear conditioning is less reliant on muscarinic signals in prelimbic cortex in females [42]. Thus, both delay and trace fear memory have sex differences in the brain processing mechanisms.

We observed no significant differences between S and SD groups of females during conditioning for both delay and trace conditioning (Fig. 2A), suggesting that an unintended significant bias due to the estrous cycle at the start of experiments is unlikely in these two groups. This was further supported by ANCOVA controlling the effect of memory acquisition as a covariate. The significant decrease of fear memory recall in the SD group occurred without significant changes in the linear regression slope (Figs. 5 and 6), suggesting that SD affects emotional memory processing while maintaining inter-individual variability in freezing levels. Because the estrous cycle can affect sleep states [43], interaction among emotional memory circuits, sleep state, and estrous cycle should be an important question in this field. A recent study in rats shows that vertex auditory responses are enhanced at the proestrus stage across sleep/wake states, particularly during REM sleep [44]. Further study is needed to understand how the estrous cycle and sleep interact in the processing of associative auditory fear memory.

### Limitations

A limitation of this study is that SD affects global brain states, making it difficult to identify specific brain regions responsible for extinction facilitation. Although SD impacts the entire brain, it did not alter general fear or anxiety levels, as evidenced by the lack of significant changes in freezing to CS- (Fig. 3A_2_; Fig. 3B_2_). SD with gentle stimulation is known not to elevate corticosterone levels [45], which can impair memory consolidation [46]. After a 6-hour SD period, rebound sleep tends to occur, especially within the first 2 hour, and natural EEG patterns resume within 8 hour [47]. To avoid overlap between rebound sleep and behavioral sessions, we implemented a 24-hour interval between conditioning on Day 1 and Extinction #1 on Day 2. Previous studies from our group and others suggest that behavioral testing at different circadian phases supports the role of post-learning sleep [48, 49]. Our findings align with these studies, showing lower freezing during post-extinction remote recall in the active phase (ZT16) compared to the inactive phase (ZT4) [50]. However, these approaches do not distinguish the roles of non-REM and REM sleep, even though fear conditioning fragments REM sleep [51] and increases non-REM sleep [52]. Although sleep-state specific optogenetic manipulations have been applied to delay fear memory [8, 9, 53], further application is required particularly for trace fear memory.

## Conclusion

Using both male and female mice, we found that post-conditioning SD rapidly enhanced trace fear extinction and gradually enhanced delay fear extinction. The advantages of auditory fear conditioning include trial-averaged or time-locked analysis of neural activity during CS and US. Further studies investigating long-term interactions among memory types, sleep, and sex differences are needed to improve our understanding of individualized emotional memory storage and resilience.

## Data Availability

The data supporting the findings of this study are available from the corresponding author on reasonable request.

## Abbreviations

ANCOVA: Analysis of Covariance
ANOVA: Analysis of Variance
CS: Conditioned Stimulus
PTSD: Post-Traumatic Stress Disorder
REM: Rapid-Eye Movement
SD: Sleep Deprivation
SEM: Standard Error of the Mean
US: Unconditioned Stimulus
ZT: Zeitgeber Time
